# Pre-Growth Culture Conditions Affect Type 1 Fimbriae-Dependent Adhesion of *Salmonella*

**DOI:** 10.3390/ijms21124206

**Published:** 2020-06-12

**Authors:** Beata Klasa, Anna Ewa Kędzierska, Krzysztof Grzymajło

**Affiliations:** 1 Department of Biochemistry and Molecular Biology, Faculty of Veterinary Medicine, Wrocław University of Environmental and Life Sciences, 50-375 Wrocław, Poland; beata.klasa@upwr.edu.pl; 2 Lukasiewicz Research Network—PORT Polish Center for Technology Development, Stablowicka 147, 54-066 Wroclaw, Poland; annae.kedzierska@gmail.com

**Keywords:** type 1 fimbriae, *Salmonella*, adhesion, phase variations, infection, growth conditions, passages

## Abstract

Among various fimbrial structures used by *Salmonella enterica* to colonize host tissues, type 1 fimbriae (T1F) are among the most extensively studied. Although some experiments have shown the importance of T1F in the initial stages of *Salmonella* infection, their exact role in the infection process is not fully known. We suggested that different outcomes of T1F investigations were due to the use of different pre-infection growth conditions for the induction of the T1F. We utilized qPCR, flow cytometry, and a wide range of adhesion assays to investigate *Salmonella* Choleraesuis and *Salmonella* Typhimurium adhesion in the context of T1F expression. We demonstrated that T1F expression was highly dependent on the pre-infection growth conditions. These growth conditions yielded T1F+ and T1F- populations of *Salmonella* and, therefore, could be a factor influencing *Salmonella-*host cell interactions. We supported this conclusion by showing that increased levels of T1F expression directly correlated with higher levels of *Salmonella* adherence to the intestinal epithelial IPEC-J2 cell line.

## 1. Introduction

*Salmonella enterica* subsp. *enterica* is a food and water-borne Gram-negative bacterial pathogen with the ability to infect a wide range of animal species, from reptiles to birds and mammals. This subspecies can cause a wide range of illnesses, from typhoid fever caused by host-restricted serovars like *Salmonella* Typhi (*S*. Typhi) or *Salmonella* Choleraesuis (*S*. Choleraesuis) to self-limiting gastroenteritis caused by many *Salmonella* serovars [1]. To date, the best-studied serovar is *Salmonella* Typhimurium (*S*. Typhimurium), which can infect many different cell types and animals and can act as a model of both gastroenteritis and systemic infection depending on the host species and experimental designs [2].

Flagella, fimbriae, and the SPI-1 T3SS (*Salmonella* pathogenicity island 1; type three secretion system) are *Salmonella’s* virulence factors expressed in a strictly defined order in various stages of bacterial infection [3,4]. After oral infection, *Salmonella* uses flagella to move to the proximity of the intestinal epithelial cells to colonize intestinal lumen, and then uses fimbriae for cell attachment and colonization of gut mucosa, and, finally, uses SPI-1 T3SS for invasion. Among 13 different fimbrial operons in the *Salmonella* genome (*agf* (*csg*), *fim*, *lpf*, *pef*, *bcf*, *stb*, *stc*, *std*, *stf*, *sth, sti*, *saf*, and *stj*), type 1 fimbriae (T1F) encoded by the *fim* operon are one of the most extensively studied (reviewed in [5]). T1F is relatively long (2 µm), rod-shaped structures composed primarily of 500 to 3000 FimA monomers [6], with a single lectin-like protein, FimH, present at the tip. FimH is directly responsible for the binding properties of T1F to mannose-containing oligosaccharides carried by surface glycoproteins of eukaryotic cells [7,8], as well as to unknown non-mannosylated receptors found in avian cells [9]. The presence of T1F has been proven to directly impact the *Salmonella* adhesion level to host epithelial cells [7,8,10]; however, regulation of *fim* cluster expression, especially during infection, still needs to be elucidated.

T1F expression is regulated by many genetic and environmental factors, including three genes—*fimW*, *fimZ*, and *fimY—*located in the *fim* operon (explained in detail in [5]). Six of the structural genes within the *fim* operon are under the control of the *fimA* promoter [11]. Some specific growth conditions can induce or inhibit T1F expression via direct or indirect activation or deactivation of the *fimA* promoter (reviewed in [5]). Growth in static liquid culture and aerobic conditions and multiple passages has led to an increase in the fraction of T1F positive bacteria, and growth on solid agar has resulted in no T1F positive population [12,13]. It was later shown that different environmental conditions influence T1F phase variation [14], and those phenomena could also be responsible for other determinants required for *Salmonella* invasion and intracellular survival [15] Despite this, laboratory conditions for the induction of T1F are still not consistent across the field. Regardless of the large number of studies on *Salmonella* T1F, there are still no detailed data regarding its expression in vitro and in vivo, including in terms of contact with the host cells.

Therefore, we decided to investigate the *Salmonella* adhesion level to IPEC-J2 cell line, a widely used intestinal epithelial cell model [16] in the context of the above-mentioned T1F inducement conditions. We measured the variation in the *fimH* gene and FimH protein expressions in different growth phases, culture conditions, and, finally, during direct contact with the eukaryotic cells. Here, we described the changes in *Salmonella fimH* gene transcription and FimH surface expression that were influenced by growth phase, serial passage, culture agitation, and contact with mammalian intestinal epithelial cells. These changes in expression levels of T1F directly correlated with the level of adhered *Salmonella* to host cells during the early stages of infection.

## 2. Results

### 2.1. Adhesion of Salmonella to IPEC-J2 Cells Depended on Pre-Infection Bacterial Growth Conditions

**Figure 1:**
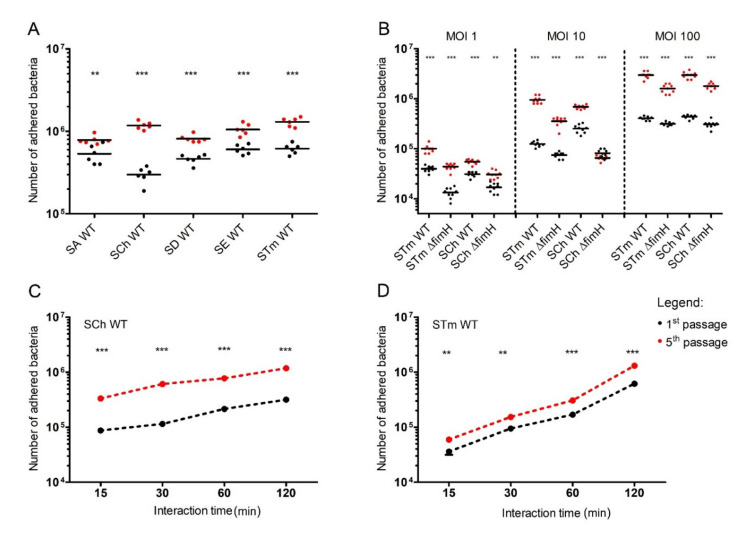
Adherence of *Salmonella* to porcine intestinal epithelial cell line IPEC-J2. (**A**) Five *Salmonella* serovars (*S*. Abortusovis (SA), *S*. Choleraesuis (SCh), *S*. Dublin (SD), *S*. Enteritidis (SE), and *S.* Typhimurium (STm)) after the first or fifth passage were incubated (multiplicity of infection (MOI) 50) for two hours with cell monolayers in a 24-well plate. (**B**) *S.* Choleraesuis (SCh WT) and *S.* Typhimurium (STm WT) wild types and their Δ*fimH* mutants (SCh Δ*fimH* and STm Δ*fimH,* respectively*)* after the first or fifth passage were incubated (MOI 1, 10, and 100) for two hours with IPEC-J2 cell monolayers. (**C**) *S.* Choleraesuis (SCh) and (**D**) *S.* Typhimurium (STm) wild types after the first or fifth passage. Statistical differences between the first (black dots) and fifth (red dost) passage were analyzed by Student’s *t*-test and are presented as individual values with a geometric mean (**A**,**B**) or as geometric mean (**C**,**D**). ** *p* < 0.01, and *** *p* < 0.001.

For analysis of how serial passages of *Salmonella* impact its adhesion ability to intestinal epithelial cells, we performed adhesion/infection tests with five different *Salmonella* serovars (Abortusovis, Choleraesuis, Dublin, Enteritidis, and Typhimurium) to IPEC-J2 intestinal epithelial cell line after the first and the fifth passage at the multiplicity of infection (MOI) of 50 (Figure 1A). We noticed that the number of adhered bacteria after the fifth passage was significantly higher (*p* < 0.01 for *S*. Abortusovis, and *p* < 0.001 for all other serovars) in every tested serovar when compared with the adhered bacteria after the first passage. However, the most profound differences were noticed for *S*. Typhimurium and *S*. Choleraesuis (*p* < 0.001). Therefore, we decided to investigate those two serovars further.

We tested the adhesion of *S*. Typhimurium and *S*. Choleraesuis and their Δ*fimH* mutants with no expression of T1F [17] using different MOI, starting from 1 to 100 (Figure 1B). In the case of both analyzed serovars, adhesion strongly increased after the fifth passage compared with the first passage in every tested MOI (Figure 1B). Starting with the lowest number of bacteria per cell (MOI 1), there was 2.5-fold more adhered *S*. Typhimurium and two-fold more of adhered *S*. Choleraesuis after the fifth passage versus the first passage. Infection with MOI 10 produced a 10-fold increase in adhered *S*. Typhimurium and a three-fold increase in adhered *S*. Choleraesuis after the fifth passage compared with the first passage. In the case of the highest MOI, the number of adhered bacteria was 10-fold higher for both analyzed serovars after the fifth passage compared to the first passage. Despite significantly lower adhesion of Δ*fimH* mutants in comparison to WT strains, in the case of bacteria without T1F, we also observed a significantly higher number of adhered bacteria after the fifth passage (Figure 1B). T1F-dependent adhesion (calculated as the difference between WT strains and Δ*fimH* strains as a percentage of a total number of the adhered bacteria) reached the highest values for MOI 10 (40–68% for the first passage, and 63–90% for the fifth passage) and its lowest values for MOI 100 (22–29% for the first passage, and 39–46% for the fifth passage).

The counts of adhered *S.* Typhimurium and *S*. Choleraesuis increased with infection time, starting from ~2.4–8 × 10^4^ bacteria after 15 min through 1.5–2.5 × 10^5^ bacteria after one hour up to 0.65–1.5 × 10^6^ after two hours (Figure 1C,D). The number of adhered bacteria after the fifth passage was approximately three to four times higher for *S*. Choleraesuis and two to three times higher for *S.* Typhimurium compared with the first passage in every tested time-point. When bacteria were grown with agitation, the overall adhesion was approximately two times lower for both serovars after the first passage but with no significant increase after the fifth passage. We observed no significant differences between the wild-type (WT) strain and the mutants after the first and fifth passages (Figure S2).

### 2.2. Salmonella FimH Expression Was Dependent on Growth Conditions and Correlated with Adhesion Level

The above-mentioned adhesion tests revealed that *S.* Typhimurium and *S.* Choleraesuis serial passages under static growth conditions increased the number of bacteria adhered to IPEC-J2 cells. Based on this, we decided to investigate how T1F is expressed in those growth conditions. As determined by T1F biogenesis, FimH protein expression is crucial for the presence of T1F at the surface of *Salmonella* [17]. Therefore, we analyzed *fimH* transcription levels via qPCR followed by measurement of FimH protein surface expression using flow cytometry.

**Figure 2:**
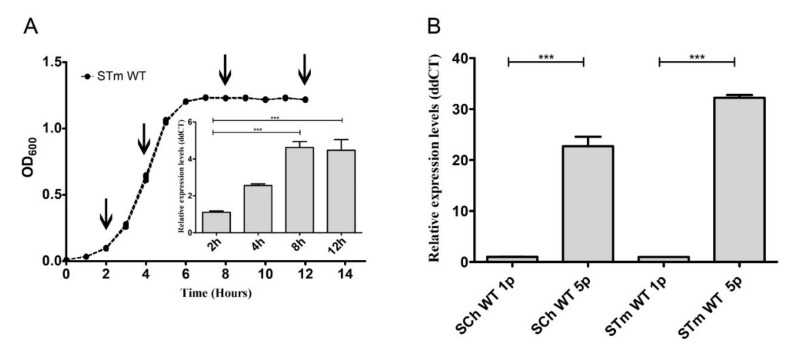
*Salmonella’s fimH* gene expression depended on growth conditions. (**A**) The growth curve of *Salmonella* Typhimurium wild type after the first passage. The values represent the mean ± SD of six independent experiments. The relative expression level of *fimH* mRNA was measured after 2, 4, 8, and 12 h after inoculation with 5 × 10^6^ bacteria. (**B**) Relative expression of *fimH* mRNA in *S.* Choleraesuis (SCh WT) and *S.* Typhimurium (STm WT) wild types after the first (1p) and the fifth (5p) passage during static growth in Luria-Bertani (LB) broth. Data represent the mean ± SD of four independent experiments. Triplicate samples were analyzed in each experiment to confirm the accuracy and reproducibility of qPCR. Statistical differences were analyzed by one-way Kruskal–Wallis test (**A**) or Student’s *t*-test (**B**) and are presented as the mean. *** *p* < 0.001.

First, growth curves in stationary growth conditions (without agitation) were obtained for *S*. Typhimurium and *S.* Choleraesuis and their Δ*fimH* mutants after the first and fifth passages. There were no differences in any of the typical growth phases, either between passages or analyzed strains (Figure S3). Therefore, we suggested that the expression of *fimH* had no impact on the growth of *Salmonella*; therefore, the presence of T1F at the surface of bacteria did not impact any of the typical growth phases. Following that observation, we decided to measure the relative expression level of *fimH* mRNA at 2, 4, 8, and 12 h after inoculation with 5 × 10^6^ bacteria. We noticed the differences in *fimH* gene expression during different growth phases of *S*. Typhimurium wild type strain (Figure 2A). After entering the exponential growth phase, *fimH* gene expression was relatively low; then, its expression increased by about 2.6 times in the mid-log phase and reached its maximum (4.5-fold; *p* < 0.001) in the early and late stationary phases. We then decided to investigate if *fimH* expression in late stationary phase differs between the first and the fifth passages and, therefore, correlates with the observed changes in adhesion levels. We observed a significant increase (around 20-fold for *S*. Choleraesuis and around 30 fold for *S*. Typhimurium) of *fimH* mRNA (*p* < 0.001) after the fifth passage compared to the first passage in both tested serovars (Figure 2B).

**Figure 3:**
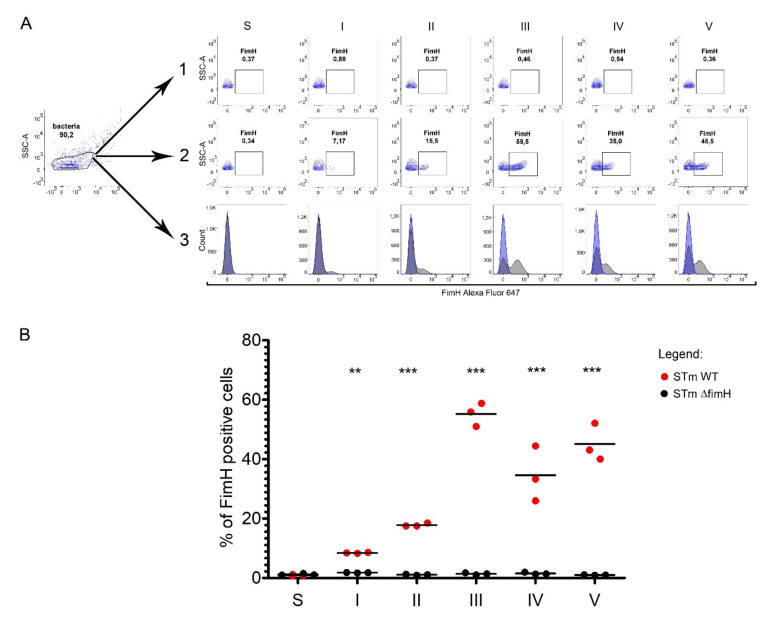
FimH protein expression on the surface of *Salmonella* Typhimurium depends on growth conditions. (**A**) Representative dot plots for the gating strategy of FimH positive: (1) STm Δ*fimH* and (2) STm WT after growth on solid agar (S) or after five serial passages (I-V) in a liquid medium without agitation. (3) Representative overlay histograms of STm WT (grey histograms) and STm Δ*fimH* (blue histograms). (**B**) Percentage of FimH positive STm Δ*fimH* (black dots) and/or STm WT (red dots) after growth on solid agar (S) or after five serial passages (I-V) in a liquid medium without agitation measured by flow cytometry. Data represent the mean ± SD of three independent experiments. Statistical differences between STm Δ*fimH* and STm WT were analyzed with Student’s *t*-test and are presented as individual values with a mean. ** *p* < 0.01, and *** *p* < 0.001.

We measured the presence of FimH protein on the surface of *S*. Typhimurium using a polyclonal antibody with flow cytometry (Figure 3A,B). The percentage of FimH-positive bacteria after every passage from the first up to the fifth was analyzed based on the gating strategy shown in Figure 3A. The number of FimH-positive cells increased with serial passages, starting with an average of 8.5% after the first passage to 18% after the second and reaching the maximum level at an average of 55% after the third passage. The fimbriation level after the fourth and the fifth passages remained in a range of 35–50%. No FimH-positive cells were observed when measuring *S*. Typhimurium cells grown on agar plates (Figure 3A,B).

### 2.3. Salmonella fimH Gene Expression Increased during Contact with IPEC-J2 Cells

**Figure 4:**
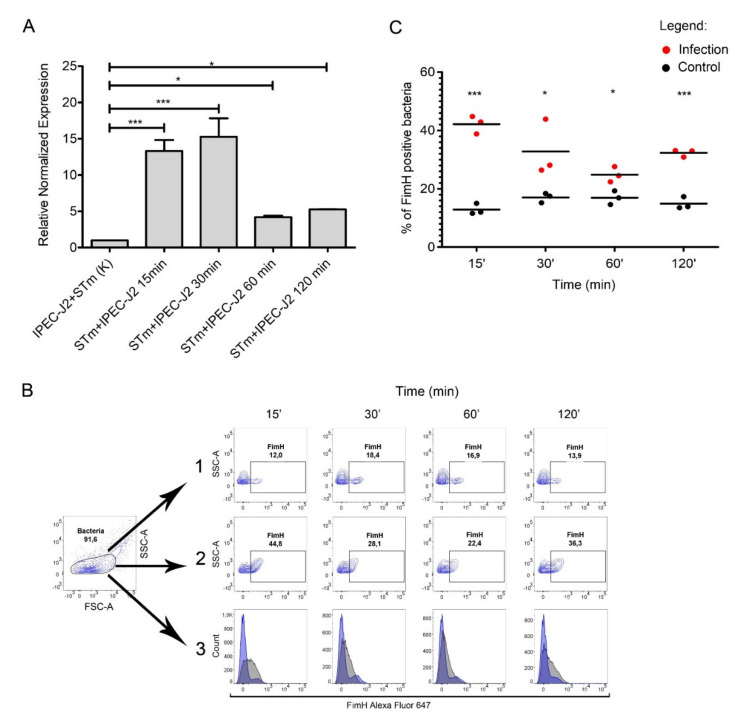
FimH expression during IPEC-J2 cells infection with *Salmonella* Typhimurium. (**A**) Relative expression of *fimH* mRNA in *S.* Typhimurium attached to IPEC-J2 cells at 0, 15, 30, 60, and 120 min after 1st passage. Data represent the mean ± SD of three independent experiments. Triplicate samples were analyzed in each experiment to confirm the accuracy and reproducibility of qPCR. Statistical differences were analyzed using one-way ANOVA and are presented as means. (**B**) Representative dot plots for the gating strategy of FimH-positive *S*. Typhimurium. (1) Control bacteria *S*. Typhimurium in cell culture medium without IPEC-J2 cells in conditions mimicked those used for the cell infection experiments. (2) *S.* Typhimurium attached to IPEC-J2 cells at 0, 15, 30, 60, and 120 min (MOI 50) after the first passage. (3) Representative overlay histograms comparing the expression of FimH protein in *S.* Typhimurium attached to IPEC-J2 cells (grey histograms) and control bacteria (blue histograms). (**C**) Percentage of FimH-positive *S.* Typhimurium attached to IPEC-J2 cells or control bacteria at 0, 15, 30, 60, and 120 min after the first passage measured by flow cytometry. Data represent the three individual values and a mean from three independent experiments. Statistical differences were analyzed by Student’s *t*-test and are presented as individual values with a mean. * *p* < 0.05, and *** *p* < 0.001.

To assess the expression of T1F by *S*. Typhimurium during infection of the IPEC-J2 cells, we performed qPCR to measure *fimH* transcription rates at 15, 30, 60, and 120 min post-infection in the bacterial population adhered to IPEC-J2 cells (Figure 4A).

We observed a significant difference in *fimH* gene expression when comparing *S.* Typhimurium adhered to IPEC-J2 versus control samples. We observed an increase in *fimH* mRNA expression throughout the infection (Figure 4A). *fimH* gene transcription in *S.* Typhimurium was up-regulated by approximately 15-fold after 15 and 30 min post-infection (*p* < 0.001), and even after one and two hours post-infection, we observed an approximately five-fold (*p* < 0.05) increase in *fimH* transcription, compared to the control RNA pool representing bacteria in a culture media mixed with IPEC-J2 cells immediately before RNA isolation. Importantly, the expression of *fimH* mRNA in bacteria not attached to the IPEC-J2 cells was less than two-fold (Figure S4A). We also observed no change in *fimH* expression when bacteria were cultured in medium without contact with IPEC-J2 cells.

To investigate whether *fimH* transcription correlates with protein present on the bacterial cell surface, we measured FimH surface protein by flow cytometry over the duration of our adhesion assay with the IPEC-J2 cell line (Figure 4B). The number of FimH-positive cells was significantly higher in bacteria attached to IPEC-J2 cells compared to control samples at each time point. After 15, 30, 60, and 120 min of infection, around 42%, 32%, 26%, and 33% of the adhered bacteria expressed FimH, respectively, and around 13%, 16%, 16%, and 14% of the control cells expressed FimH, respectively (Figure 4A,B). Similarly, only about 17% of *Salmonella* Typhimurium from the medium above the cells after two hours of infection expressed FimH on the bacterial surface (Figure S4B).

## 3. Discussion

*Salmonella enterica* uses various factors for successful infection by the fecal-oral route. The SPI-1 T3SS is crucial for *Salmonella* infection, but other systems, including flagella and fimbrial operons, contribute to *Salmonella* pathogenesis [18,19,20,21,22,23].

Many experiments support T1F use in the first stages of the infection process, mostly based on in vitro studies of contact between cultured cell lines and various *Salmonella* serovars and mutants [7,10,24]. Some reports exclude T1F in adhesion to certain intestinal epithelial cells [25,26,27]. One of the possible reasons for this disagreement regarding the role of T1F in the host-pathogen interaction could be the different bacterial culture conditions that influence T1F expression. Expression of *Salmonella* virulence factors, including T1F, is regulated by many environmental signals during the infection process. Since the growth conditions of bacteria during in vitro experiments could alter the expression of virulence factors, researchers should grow bacteria in conditions that induce the expression of their system of interest. The expression of fim cluster genes is favored in static liquid medium [12,13], and growth on solid agar inhibits T1F expression. In the above-mentioned T1F-inducing conditions, *Salmonella* cultures have contained cells in two phenotypic states—T1F+ and T1F- [15,28]. Serial passages have been suggested to increase the T1F+ population [12,13]. Despite this, laboratory conditions for the induction of T1F are still not consistent across the field. Therefore, in this work, we attempted to answer in detail how the pre-invasion growth conditions affect the adhesion properties of *Salmonella* in the context of T1F expression.

Based on the strategy previously described by Duguid et al. [12,13], our previous studies on adhesion mediated by T1F used bacteria after the fifth passage in static growth conditions [7,8,22,23]. In this study, we decided to test the importance of these growth conditions in the role of T1F during adhesion to intestinal epithelial cells. We compared selected *Salmonella* strains from five different serovars after the first and fifth passages in static LB medium by measuring their adhesion levels to porcine intestinal epithelial cell line IPEC-J2 [7,29]. The IPEC-J2 cell line used in our study is a widely accepted, non-transformed epithelial cell line model that supports interaction with a variety of bacterial species, including different *Salmonella* serovars [8,16]. For every tested serovar, serial passages increased the percentage of adhered bacteria significantly, with the most striking differences observed in *Salmonella enterica* serovars—Choleraesuis and Typhimurium. Based on this, these two serovars with different host specificity and different abilities for binding of eukaryotic cells [30] were selected for further investigation. After the first passage, bacteria adhered more weakly compared to bacteria after the fifth passage for both tested serovars, regardless of MOI used. This pattern was true even for Δ*fimH* mutants without T1F expression despite the lower number of adhered bacteria when compared with WT strains. This suggested that different structures were involved in adhesion, which was also induced by serial passages without agitation since a large number of different adhesive structures can impact *Salmonella* adhesion ability [31,32]. However, among 13 identified fimbrial operons found in *S*. Typhimurium [33], except for T1F, few are found to be expressed in vivo or after infection of ligated ileal loops [18,34]; only curli fimbriae, long polar fimbriae, and plasmid-encoded fimbriae (Pef) are expressed in vitro under standard laboratory conditions [31,33,34,35].

From those, Pef is reported to be expressed in static conditions and an acidic medium [36]. T1F expression has been reported to prevent the expression of Pef [37]; therefore, a lack of T1F may induce Pef expression. On the other hand, also SPI-1 T3SS has been shown to be activated in *S*. Typhimurium in low oxygen and stationary growth phase [32].

Those observations were confirmed by adhesion/infection assays using pre-infection growth with agitation. Adhesion levels were low despite high MOI, and there were no significant differences between WT strain and *fimH* null mutants after the first or fifth passages (Figure S2). Bearing that in mind, T1F-dependent adhesion—defined as a difference in adhesion between WT and Δ*fimH* strains—was significantly lower in both serovars after the first passage compared with after the fifth passage. Under relatively high MOI (100) and after the first passage without agitation (the experimental conditions frequently used by convenience), T1F-dependent adhesion was below 30% for both serovars. In general, moderate MOI, in the range of 10–50, was found to be the most prominent in the case of the T1F role in infection experiments. The above-mentioned experiments showed that the growth of *Salmonella* in our T1F-inducing conditions involved a drastic increase in adhesion levels. This raised questions about the exact T1F expression level and timing of its expression. Therefore, we were interested if T1F expression on mRNA and protein level would confirm our findings regarding the impact of growth conditions on adhesion assays. We found that during static growth, *fimH* mRNA expression was significantly higher in the early and late stationary phases than in the logarithmic growth phase. When we compared *fimH* mRNA expression after the first and fifth passages in the above-mentioned conditions, expression after the fifth passage was significantly up-regulated. We noticed significant differences in *S*. Typhimurium fimbriation levels between the first and subsequent passages. This indicated that, at least in the case of *S.* Typhimurium T1F experiments, bacteria should be used after, at least, the third passage.

Initial contact with host cells is frequently a crucial phase in bacterial infection. Many studies show T1F-dependent adhesion to cell lines originating from intestinal epithelial cells [5,7,22,23]. However, some reports show no contribution of T1F to the adhesion to those cell types [25,26]. Given the data reported here, we speculated that these differing conclusions reported in the literature might arise from the use of different pre-infection growth conditions for *Salmonella*. To further investigate this observation, we decided to infect the IPEC-J2 cell line with *S.* Typhimurium after the first passage in static conditions, a condition that induced relatively low T1F expression (Figure 3B). We noticed that the attached bacteria transcribed a significantly higher amount of *fimH* mRNA compared to control cells. Similarly, a higher percentage of the attached *S*. Typhimurium population was T1F-positive compared with the control cells. These results suggested that, during infection, the adhered T1F+ population further increased T1F expression and that bacteria, which adhered at the later time-points, might be a subpopulation of this initially adhered surface-associated T1F+ bacteria. We speculated that this might be a division of *Salmonella* into different subpopulations during infection; this was in agreement with our observations that during infection, unattached *S.* Typhimurium cells did not increase *fimH* transcription or T1F surface expression. In *Salmonella*, there are three major regulatory proteins: FimZ, FimY, and FimW. The first two activate T1F expression, whereas FimW is a negative regulator [4]. Since T1F expression can be regulated by many environmental signals, like pH or ions availability, as well as indirectly by factors involved in metabolism or stress response, we only speculated whether direct contact with the host cells activates FimZ or FimY or deactivate FimW and if this is direct interaction or activation by a signal-transducing cascade. Considering that, the exact reasons why *S*. Typhimurium exhibits a highly distinct expression of T1F-dependent on its localization during infection remains to be elucidated.

In conclusion, we analyzed T1F expression by *S*. Choleraesuis and *S*. Typhimurium growing under T1F-inducing conditions, as well as during the first stages of the interaction with the host cells. We demonstrated that T1F expression was highly dependent on the pre-infection growth conditions, dividing *Salmonella* into T1F+ and T1F-populations, and could be a factor influencing the outcome of *Salmonella-*host cell interactions in vitro. T1F surface expression directly correlated with the level of *Salmonella* adhesion to the intestinal epithelial IPEC-J2 cell line. Additionally, the IPEC-J2-adhered *Salmonella* population was significantly richer in T1F+ bacteria, which might suggest a further specialization of the T1F+ population. Finally, we clarified that the pre-experimental growth conditions used to study *Salmonella* T1F function should be chosen with an understanding of the impacts of those culture conditions on experimental outcomes, rather than using culture conditions based on convention.

## 4. Materials and Methods

### 4.1. Bacterial Strains and Culture Conditions

All bacterial strains, plasmids, and oligonucleotides used in this study are described in Table 1, Table 2, and Table 3, respectively. When necessary, antibiotics were used at the following concentrations: ampicillin at 100 µg/mL, streptomycin at 50 µg/mL, kanamycin at 40 µg/mL, and gentamycin at 20 µg/mL. For adhesion/infection tests and *fimH* gene expression tests, all strains were cultured in static conditions (without agitation) at 37 °C in Luria-Bertani (LB) broth unless stated otherwise or on LB-agar plates. Bacteria were passaged every 24 h from the moment of inoculation in the amount of 10^7^ cell/mL to fresh medium. For all assays, bacteria were grown for 12 h before the experiment.

### 4.2. Cells and Cell Culture

The porcine intestinal epithelial IPEC-J2 cells [29] used in adhesion/infection and infection assays were cultured at 37 °C with 5% CO_2_ in Dulbecco’s Modified Eagle Medium (DMEM), as described previously [7], and were supplemented with 10% fetal calf serum (FCS, Invitrogen, Carlsbad, CA, USA) and 2 mM glutamine.

### 4.3. Construction of Strains and Plasmids

For all cloning procedures, PCR was performed with Q5 High-Fidelity DNA Polymerase (New England Biolabs M0491S, Ipswich, MA, USA) according to the manufacturer’s instructions. To construct the Δ*fimH* suicide plasmid, the DNA fragments (800 bp) flanking the chromosome regions of interest were PCR amplified using CG_F, ∆FIMH_B, ∆FIMH_D, and CG_R primers, fused by overlap extension PCR and inserted into the pEMG suicide plasmid (Figure S1). The knock-out of the *fimH* gene in *S.* Typhimurium was based on the method previously described by Martínez-García [39]. Briefly, Δ*fimH* pEMG was mobilized from S17-1 λpir into the recipient *S*. Typhimurium SL1344 strain by conjugation. *S.* Typhimurium transconjugants clones that had integrated the suicide plasmid by homologous recombination were selected on solid minimal medium M9 supplemented with 0.2% glucose and 50 µg/mL of kanamycin. Positive clones were transformed with the pSW-2 plasmid and stimulated on LB agar with gentamicin and m-Toluate to induce I-SceI expression and provoking cleavages of the chromosome at the inserted I-SceI restriction sites [39,40]. The transformants that lost integrated pEMG were identified by their kanamycin sensitive phenotype. The absence of the *fimH* gene was confirmed by PCR using the primers F_seq and R_seq (Table 3) and sequencing. Finally, the unstable pSW-2 was eliminated from the clones of interest by two passages in LB.

### 4.4. Determination of the Growth Curves

The growth curves of *S.* Choleraesuis and *S.* Typhimurium strains were determined for every passage. Briefly, bacteria (5 × 10^6^ colony-forming units (CFU)) after every passage were inoculated in fresh LB broth and cultured without shaking at 37 °C for 12 consecutive hours. OD_600_ was measured every hour using a UV-vis spectrophotometer (BioRad, Hercules, CA, USA). Each measurement was repeated at least three times and in triplicate. After 2, 4, 8, and 12 h, bacteria were harvested and lysed with fenosol as the first step for RNA extraction.

### 4.5. Adhesion/Infection Assays

*Salmonella* isolates were washed in PBS after the first or fifth passages, resuspended in the cell culture medium, and were then adjusted by dilution to provide appropriate MOI (multiplicity of infection) of bacteria to host cells in culture wells of a 24-well plate (Greiner, Frickenhausen, Germany). Confluent monolayers of IPEC-J2 cells were infected for 15, 30, 60, or 120 min. After incubation, cells were washed three times with PBS and lysed with PBS containing 0.1% Triton X-100 (Sigma-Aldrich, Saint Luis, MO, USA) for 10 min. Bacterial suspensions were serially diluted with PBS, plated on LB-agar plates, and incubated overnight at 37 °C, followed by CFU (colony-forming unit) calculation.

### 4.6. Infection Experiments

*Salmonella* isolates, after the first passage, were washed with PBS, resuspended in the cell culture medium, and adjusted by dilution to provide appropriate MOI. Confluent monolayers of IPEC-J2 cells were infected for 15, 30, 60, or 120 min at 37 °C with 5% CO_2_. After incubation, cells were washed with PBS, then detached mechanically and centrifuged at 2000× *g* for 5 min. Pellets were lysed with fenosol as the first step for RNA extraction. Alternatively, after the incubation and washing steps, cells were lysed with 0.1% Triton X-100 (Sigma) for 10 min, diluted to 10 × 10^6^ bacterial cells in staining buffer (PBS + 0.5% Bovine serum albumin (BSA)), and then the procedures described for flow cytometry were applied. Control samples from in vitro-grown bacteria were obtained by incubation of bacteria in cell culture medium without IPEC-J2 cells in conditions that mimicked those used for the cell infection experiments or by mixing IPEC-J2 cells with the appropriate number of bacteria immediately before RNA isolation in qPCR infection control.

### 4.7. Quantitative PCR (qPCR)

Total bacterial RNA was extracted from 2 mL of bacterial culture or infected cells using the Total RNA Mini Plus (AABiotech, Gdynia, Poland) according to the manufacturer’s instructions. Residual DNA was digested for 30 min at 37 °C in a total volume of 20 µL using DNase I (AABiotech). Total RNA was quantified by A260 measurements (DS-11 FX; DeNovix), verified by agarose gel electrophoresis, and diluted to 1 µg/µL before cDNA synthesis. First-strand cDNA was synthesized using the iScript (Bio-Rad) according to the manufacturer’s instructions. The relative amounts of mRNA were quantified by qPCR using the CFX thermocycler (Bio-Rad) and EvaGreen (IMMUNIQ). The reaction mixture contained 10x polymerase buffer without magnesium (Pol Buffer A; EURx), 2.5 mM MgCl_2_, 5 mM dNTPs, 20x EvaGreen Dye, 2.5 units of Optitaq DNA Polymerase (EURx), and 0.1 mM of each primer. cDNA was amplified as follows: 5 min incubation at 95 °C for initial denaturation, followed by 35 cycles of 20 s denaturation at 95 °C, 20 s annealing at 56 °C, and 15 s elongation at 72 °C. The target gene was normalized using 16S RNA as a reference. The comparative Cq method was used for the relative quantification of gene expression. All experiments were performed at least three times, and triplicate samples were analyzed in each experiment to confirm the accuracy and reproducibility of the qPCR.

### 4.8. Flow Cytometry

A total of 10 × 10^6^ bacteria were stained with anty-FimH rabbit polyclonal antibody [41] at 4 °C for 90 min in the dark. The cells were then washed twice with staining buffer and stained for 30 min at 4 °C in the dark with 0.05 µg of donkey anti-rabbit IgG Alexa Fluor 647 (clone Poly4064, Biolegend, San Diego, CA, USA). The cells were washed twice with staining buffer, and cellular fluorescence was immediately measured on the BD FACSCanto™ II cell analyzer (Becton Dickinson, Franklin Lakes, NJ, USA). A total of 20,000 events of the bacterial population (gated on Forward Scatter (FSC)-A versus Side Scatter (SSC)-A dot plots) were recorded with a rate of 600–800 events per second. Cytometer Setup and Tracking Beads (CS&T Research Beads, Becton Dickinson, USA) were used for automated quality assurance and control of machine performance. The analyses were conducted using FlowJo™ Software Version 10.6.2 (Becton Dickinson, USA).

### 4.9. Statistical Analysis

All statistical calculations were performed in GraphPad Prism (GraphPad Software, Inc., La Jolla, CA, USA). Data distribution was assessed using the Shapiro–Wilk normality test. Student’s *t*-test (parametric), one-way ANOVA (parametric), or the Kruskal–Wallis test (nonparametric) with Dunn’s multiple comparison post hoc test were performed according to the data distribution. All data collected in this study were obtained from at least three independent experiments for each condition. A *p*-value of less than 0.05 was considered statistically significant. Data are presented as the means ± standard deviation (SD). * *p* < 0.05, ** *p* < 0.01, and *** *p* < 0.001.

## Figures and Tables

**Table 1:** Bacterial strains used in this study.

Strains	Strain Tag	Characteristic	Reference
*S.* Choleraesuis Δ*fimH*	SCΔ*fimH*	*S*. Choleraesuis 6150 with *fimH gene* knockout	[7]
*S*. Typhimurium	WT	SL1344	Monack lab strain collection [38]
*S*. Typhimurium SL1344_∆*fimH*	∆*fimH*	*S*. Typhimurium SL 1344 with *fimH gene* knockout	This study
*S*. Abortusovis	520	Wild type, fimbriate	[8]
*S*. Dublin	508	Wild type, fimbriate	[8]
*S*. Enteritidis	327	Wild type, fimbriate	[8]
*E*. *coli* DH5α λpir			Monack lab strain collection
*E*. *coli* S17-1 *λpir*			Monack lab strain collection

**Table 2:** Bacterial plasmids used in this study.

Plasmid	Characteristic	Reference
pEMG	Suicide plasmid; KmR	[39]
pSW-2	Plasmid for m-toluate-inducible expression of the I-SceI enzyme; GmR	[39]
pEMG	∆*fimH*	This study

**Table 3:** Primers used for quantitative real-time PCR (qPCR) and for mutant construction.

FIMH_F	TACAGCGGCAAAGTGGAAGT
FIMH_R	GCCCCCGCCTGACTAAATAA
16S RNA_F	CAGAAGAAGCACCGGCTAAC
16S RNA_R	GCGCTTTACGCCCAGTAATT
CG_F	CGGAATTCGCCACGTTTAACGCCAACCGCAACCG
CG_R	CGGATCCCGTACCCCCAAAGGCGGGCAC
∆FIMH_B	CATTATGCCTCCCTCTATTTTTCCTTTTATGACGCCGGACG
∆FIMH_D	GGAAAAATAGAGGGAGGCATAATGATCCTTCGGCGCG
rpoD_F	ACATGGGTATTCAGGTAATGGAAG
rpoD_R	CGGTGCTGGTGGTATTTTCA
F_seq	GGCGATTACGATAGCCAGCGC
R_seq	CAGCGGGCTGAACAAAACACAAC

## Supplementary Materials

The following are available online at https://www.mdpi.com/1422-0067/21/12/4206/s1, Figure S1: Schematic overview of Δ*fimH* suicide plasmid and *Salmonella* Typhimurium Δ*fimH* strain. DNA fragments A and B (800 bp) flanking the chromosome regions of interest were PCR amplified using CG_F, ∆FIMH_B, ∆FIMH_D, and CG_R primers, fused by overlap extension PCR (fragment D) and inserted into the pEMG suicide plasmid. Figure S2: Adherence of *Salmonella* to porcine intestinal epithelial cell line IPEC-J2. *S.* Choleraesuis (SCh WT) and *S.* Typhimurium (STm WT) wild types and their Δ*fimH* mutants (SCh Δ*fimH* and STm Δ*fimH*, respectively) after 1st or 5th passage with agitation were incubated (MOI 50) for 2 h with cell monolayers in a 24-well plate. Statistical differences between first (black dots) and fifth (red dost) passage were analyzed by Student’s *t*-test and are presented as individual values with a geometric mean (**A**,**B**) or as geometric mean (**C**,**D**). * *p* < 0.05, ** *p* < 0.01, and *** *p* < 0.001. Figure S3: The growth curves of *Salmonella* Typhimurium, *Salmonella* Typhimurium Δ*fimH*, *Salmonella* Choleraesuiss, and *Salmonella* Choleraesuis Δ*fimH* after the 1st and the 5th passage. The bacterial densities in LB liquid medium were determined by measuring the absorbance every hour. Each point represents the mean value of three independent experiments. Figure S4: (**A**) Relative expression of *fimH* mRNA in *Salmonella* Typhimurium in supernatant above IPEC-J2 cells after 120 min of infection. Real-time RT-PCR was used to analyze the expression of *fimH* mRNA, and its levels were normalized against 16s RNA, and fold change was measured over the control infection. Data represent the mean ± SD of three independent experiments. Triplicate samples were analyzed in each experiment to confirm the accuracy and reproducibility of qPCR. Statistical differences were analyzed by the Student’s *t*-test. * *p* < 0.05, ** *p* < 0.01, and *** *p* < 0.001. (**B**) Percent of FimH-positive *Salmonella* Typhimurium in supernatant above IPEC-J2 cells after 120 min of infection measured by flow cytometry. Data represent the three individual values and a mean from three independent experiments. Statistical differences were analyzed by the Student *t*-test. * *p* < 0.05, ** *p* < 0.01, and *** *p* < 0.001.

## Abbreviations

T1F	Type 1 fimbriae
T3SS	Type three secretion system
SPI-1	*Salmonella* pathogenicity island 1
MOI	Multiplicity of infection

## References

[1] Uzzau S., Brown D.J., Wallis T., Rubino S., Leori G., Bernard S., Casadesus J., Platt D.J., Olsen J.E. (2000). Host adapted serotypes of *Salmonella enterica*. Epidemiol. Infect..

[2] Tsolis R.M., Xavier M.N., Santos R.L., Baumler A.J. (2011). How to become a top model: Impact of animal experimentation on human Salmonella disease research. Infect. Immun..

[3] Saini S., Slauch J.M., Aldridge P.D., Rao C.V. (2010). Role of cross talk in regulating the dynamic expression of the flagellar Salmonella pathogenicity island 1 and type 1 fimbrial genes. J. Bacteriol..

[4] Saini S., Pearl J.A., Rao C.V. (2009). Role of FimW, FimY, and FimZ in regulating the expression of type i fimbriae in *Salmonella enterica* serovar Typhimurium. J. Bacteriol..

[5] Kolenda R., Ugorski M., Grzymajlo K. (2019). Everything You Always Wanted to Know About Salmonella Type 1 Fimbriae, but Were Afraid to Ask. Front. Microbiol..

[6] Hahn E., Wild P., Hermanns U., Sebbel P., Glockshuber R., Haner M., Taschner N., Burkhard P., Aebi U., Muller S.A. (2002). Exploring the 3D molecular architecture of Escherichia coli type 1 pili. J. Mol. Biol..

[7] Grzymajlo K., Ugorski M., Suchanski J., Kedzierska A.E., Kolenda R., Jarzab A., Biernatowska A., Schierack P. (2017). The Novel Type 1 Fimbriae FimH Receptor Calreticulin Plays a Role in Salmonella Host Specificity. Front. Cell. Infect. Microbiol..

[8] Grzymajlo K., Ugorski M., Kolenda R., Kedzierska A., Kuzminska-Bajor M., Wieliczko A. (2013). FimH adhesin from host unrestricted Salmonella Enteritidis binds to different glycoprotein ligands expressed by enterocytes from sheep, pig and cattle than FimH adhesins from host restricted Salmonella Abortus-ovis, Salmonella Choleraesuis and Salmonella Dublin. Vet. Microbiol..

[9] Guo A., Cao S., Tu L., Chen P., Zhang C., Jia A., Yang W., Liu Z., Chen H., Schifferli D.M. (2009). FimH alleles direct preferential binding of Salmonella to distinct mammalian cells or to avian cells. Microbiology.

[10] Kisiela D.I., Chattopadhyay S., Libby S.J., Karlinsey J.E., Fang F.C., Tchesnokova V., Kramer J.J., Beskhlebnaya V., Samadpour M., Grzymajlo K. (2012). Evolution of *Salmonella enterica* virulence via point mutations in the fimbrial adhesin. PLoS Pathog..

[11] Purcell B.K., Pruckler J., Clegg S. (1987). Nucleotide sequences of the genes encoding type 1 fimbrial subunits of Klebsiella pneumoniae and Salmonella typhimurium. J. Bacteriol..

[12] Old D.C., Duguid J.P. (1970). Selective outgrowth of fimbriate bacteria in static liquid medium. J. Bacteriol..

[13] Duguid J.P., Anderson E.S., Campbell I. (1966). Fimbriae and adhesive properties in Salmonellae. J. Pathol. Bacteriol..

[14] Silverman M., Zieg J., Hilmen M., Simon M. (1979). Phase variation in Salmonella: Genetic analysis of a recombinational switch. Proc. Natl. Acad. Sci. USA.

[15] Brosnahan A.J., Brown D.R. (2012). Porcine IPEC-J2 intestinal epithelial cells in microbiological investigations. Vet. Microbiol..

[16] Zeiner S.A., Dwyer B.E., Clegg S. (2012). FimA, FimF, and FimH are necessary for assembly of type 1 fimbriae on *Salmonella enterica* serovar Typhimurium. Infect. Immun..

[17] Weening E.H., Barker J.D., Laarakker M.C., Humphries A.D., Tsolis R.M., Baumler A.J. (2005). The *Salmonella enterica* serotype Typhimurium lpf, bcf, stb, stc, std, and sth fimbrial operons are required for intestinal persistence in mice. Infect. Immun..

[18] Dibb-Fuller M.P., Allen-Vercoe E., Thorns C.J., Woodward M.J. (1999). Fimbriae- and flagella-mediated association with and invasion of cultured epithelial cells by Salmonella enteritidis. Microbiology.

[19] Collazo C.M., Galan J.E. (1996). Requirement for exported proteins in secretion through the invasion-associated type III system of Salmonella typhimurium. Infect. Immun..

[20] Kaniga K., Tucker S., Trollinger D., Galan J.E. (1995). Homologs of the Shigella IpaB and IpaC invasins are required for Salmonella typhimurium entry into cultured epithelial cells. J. Bacteriol..

[21] Kuzminska-Bajor M., Grzymajlo K., Ugorski M. (2015). Type 1 fimbriae are important factors limiting the dissemination and colonization of mice by Salmonella Enteritidis and contribute to the induction of intestinal inflammation during Salmonella invasion. Front. Microbiol..

[22] Kuzminska-Bajor M., Kuczkowski M., Grzymajlo K., Wojciech L., Sabat M., Kisiela D., Wieliczko A., Ugorski M. (2012). Decreased colonization of chicks by *Salmonella enterica* serovar Gallinarum expressing mannose-sensitive FimH adhesin from *Salmonella enterica* serovar Enteritidis. Vet. Microbiol..

[23] Hancox L.S., Yeh K.S., Clegg S. (1997). Construction and characterization of type 1 non-fimbriate and non-adhesive mutants of Salmonella typhimurium. FEMS Immunol. Med Microbiol..

[24] Kolenda R., Burdukiewicz M., Schiebel J., Rodiger S., Sauer L., Szabo I., Orlowska A., Weinreich J., Nitschke J., Bohm A. (2018). Adhesion of Salmonella to Pancreatic Secretory Granule Membrane Major Glycoprotein GP2 of Human and Porcine Origin Depends on FimH Sequence Variation. Front. Microbiol..

[25] Rajashekara G., Munir S., Alexeyev M.F., Halvorson D.A., Wells C.L., Nagaraja K.V. (2000). Pathogenic role of SEF14, SEF17, and SEF21 fimbriae in *Salmonella enterica* serovar enteritidis infection of chickens. Appl. Environ. Microbiol..

[26] Baumler A.J., Tsolis R.M., Heffron F. (1996). Contribution of fimbrial operons to attachment to and invasion of epithelial cell lines by Salmonella typhimurium. Infect. Immun..

[27] Patterson S.K., Borewicz K., Johnson T., Xu W., Isaacson R.E. (2012). Characterization and differential gene expression between two phenotypic phase variants in *Salmonella enterica* serovar Typhimurium. PLoS ONE.

[28] Isaacson R.E., Argyilan C., Kwan L., Patterson S., Yoshinaga K. (1999). Phase variable switching of in vivo and environmental phenotypes of Salmonella typhimurium. Adv. Exp. Med. Biol..

[29] Schierack P., Nordhoff M., Pollmann M., Weyrauch K.D., Amasheh S., Lodemann U., Jores J., Tachu B., Kleta S., Blikslager A. (2006). Characterization of a porcine intestinal epithelial cell line for in vitro studies of microbial pathogenesis in swine. Histochem. Cell Biol..

[30] Yue M., Han X., De Masi L., Zhu C., Ma X., Zhang J., Wu R., Schmieder R., Kaushik R.S., Fraser G.P. (2015). Allelic variation contributes to bacterial host specificity. Nat. Commun..

[31] Wagner C., Hensel M. (2011). Adhesive mechanisms of *Salmonella enterica*. Adv. Exp. Med. Biol..

[32] Ibarra J.A., Knodler L.A., Sturdevant D.E., Virtaneva K., Carmody A.B., Fischer E.R., Porcella S.F., Steele-Mortimer O. (2010). Induction of Salmonella pathogenicity island 1 under different growth conditions can affect Salmonella-host cell interactions in vitro. Microbiology.

[33] McClelland M., Sanderson K.E., Spieth J., Clifton S.W., Latreille P., Courtney L., Porwollik S., Ali J., Dante M., Du F. (2001). Complete genome sequence of *Salmonella enterica* serovar Typhimurium LT2. Nature.

[34] Humphries A.D., Raffatellu M., Winter S., Weening E.H., Kingsley R.A., Droleskey R., Zhang S.P., Figueiredo J., Khare S., Nunes J. (2003). The use of flow cytometry to detect expression of subunits encoded by 11 *Salmonella enterica* serotype Typhimurium fimbrial operons. Mol. Microbiol..

[35] Nuccio S.P., Baumler A.J. (2007). Evolution of the chaperone/usher assembly pathway: Fimbrial classification goes Greek. Microbiol. Mol. Biol. Rev..

[36] Hurtado-Escobar G.A., Grepinet O., Raymond P., Abed N., Velge P., Virlogeux-Payant I. (2019). H-NS is the major repressor of Salmonella Typhimurium Pef fimbriae expression. Virulence.

[37] Sterzenbach T., Nguyen K.T., Nuccio S.P., Winter M.G., Vakulskas C.A., Clegg S., Romeo T., Baumler A.J. (2013). A novel CsrA titration mechanism regulates fimbrial gene expression in Salmonella typhimurium. Embo J..

[38] Jacobson A., Lam L., Rajendram M., Tamburini F., Honeycutt J., Pham T., Van Treuren W., Pruss K., Stabler S.R., Lugo K. (2018). A Gut Commensal-Produced Metabolite Mediates Colonization Resistance to Salmonella Infection. Cell Host Microbe.

[39] Martinez-Garcia E., de Lorenzo V. (2011). Engineering multiple genomic deletions in Gram-negative bacteria: Analysis of the multi-resistant antibiotic profile of Pseudomonas putida KT2440. Environ. Microbiol..

[40] Owen S.V., Wenner N., Canals R., Makumi A., Hammarlof D.L., Gordon M.A., Aertsen A., Feasey N.A., Hinton J.C. (2017). Characterization of the Prophage Repertoire of African Salmonella Typhimurium ST313 Reveals High Levels of Spontaneous Induction of Novel Phage BTP1. Front. Microbiol..

[41] Kisiela D., Sapeta A., Kuczkowski M., Stefaniak T., Wieliczko A., Ugorski M. (2005). Characterization of FimH adhesins expressed by *Salmonella enterica* serovar Gallinarum biovars Gallinarum and Pullorum: Reconstitution of mannose-binding properties by single amino acid substitution. Infect. Immun..

