# Supplementary material for: Pre-Growth Culture Conditions Affect Type 1 Fimbriae-Dependent Adhesion of Salmonella
Source: Int J Mol Sci. 2020 Jun 12;21(12):4206. doi: 10.3390/ijms21124206 (PMC7352897; doi:10.3390/ijms21124206)
Supplement: Supplementary file 1 [file ijms-21-04206-s001.zip › Supplementary_Figure 2.pdf]

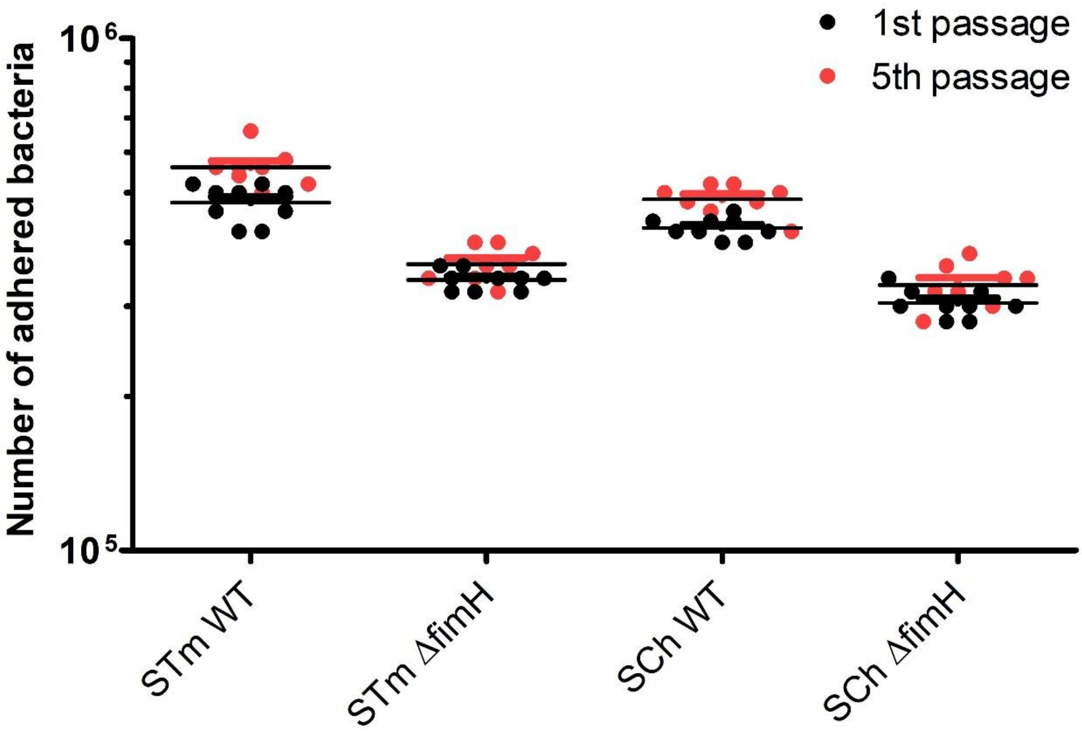

Supplementary figure 2: Adherence of *Salmonella* to porcine intestinal epithelial cell line IPEC-J2. *S.* Choleraesuis (SCh WT), and *S.* Typhimurium (STm WT) wild types and their *ΔfimH* mutants (SCh *ΔfimH* and STm *ΔfimH* respectively*)* after 1st or 5th passage with agitation were incubated (MOI 50) for 2 hours with cell monolayers in 24-well plate. Statistical differences between 1st (black dots) and 5th (red dost) passage were analyzed by t-Student test are presented as individual values with a geometric mean (A, B) or as geometric mean (C, D). *$p < 0.05$, **$p < 0.01$, and ***$p < 0.001$.
