# Supplementary material for: Pre-Growth Culture Conditions Affect Type 1 Fimbriae-Dependent Adhesion of Salmonella
Source: Int J Mol Sci. 2020 Jun 12;21(12):4206. doi: 10.3390/ijms21124206 (PMC7352897; doi:10.3390/ijms21124206)
Supplement: Supplementary file 1 [file ijms-21-04206-s001.zip › Supplementary_Figure 3.pdf]

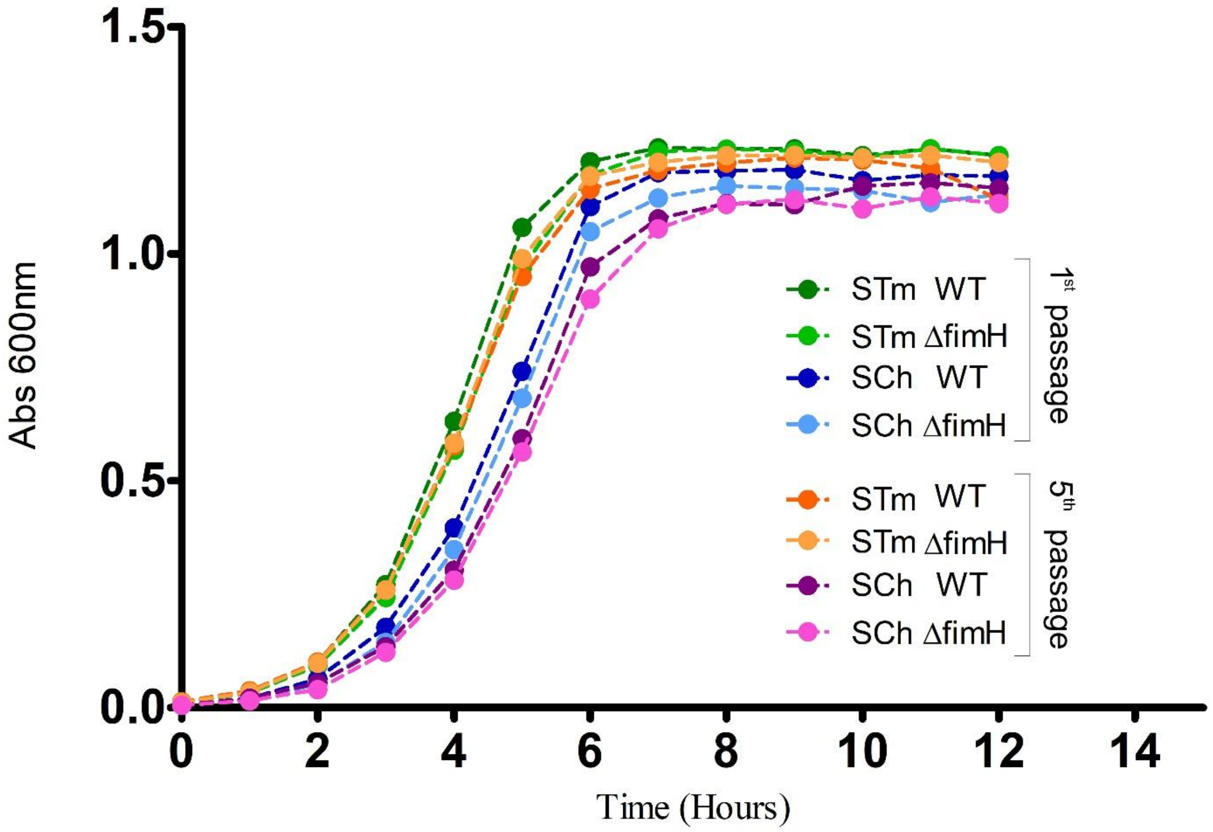

Supplementary figure 3: The growth curves of *Salmonella* Typhimurium, *Salmonella* Typhimurium *ΔfimH*, *Salmonella* Choleraesuis and *Salmonella* Choleraesuis *ΔfimH* after the 1st and the 5th fifth passage. The bacterial densities in LB liquid medium were determined by measuring the absorbance every hour. The values represent the mean with SD of three independent experiments.
