# Supplementary material for: Pre-Growth Culture Conditions Affect Type 1 Fimbriae-Dependent Adhesion of Salmonella
Source: Int J Mol Sci. 2020 Jun 12;21(12):4206. doi: 10.3390/ijms21124206 (PMC7352897; doi:10.3390/ijms21124206)
Supplement: Supplementary file 1 [file ijms-21-04206-s001.zip › Supplementary_Figure 4.pdf]

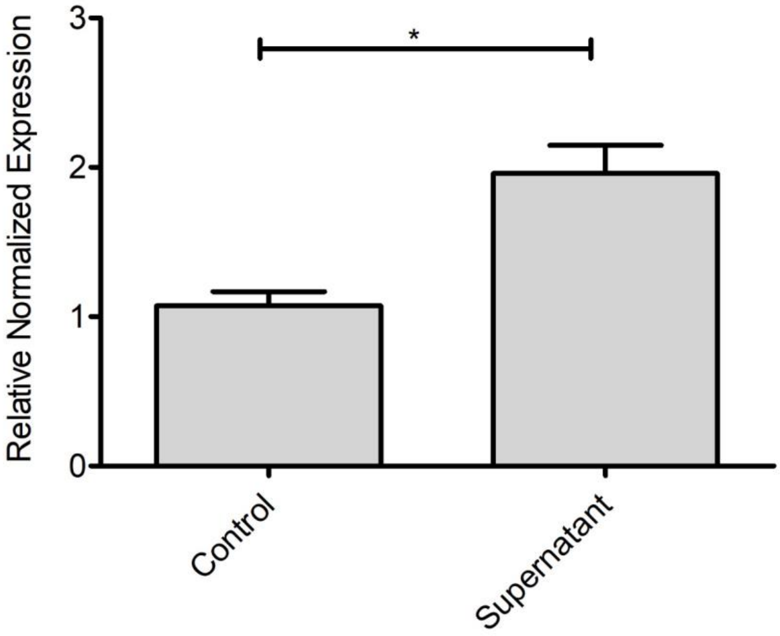

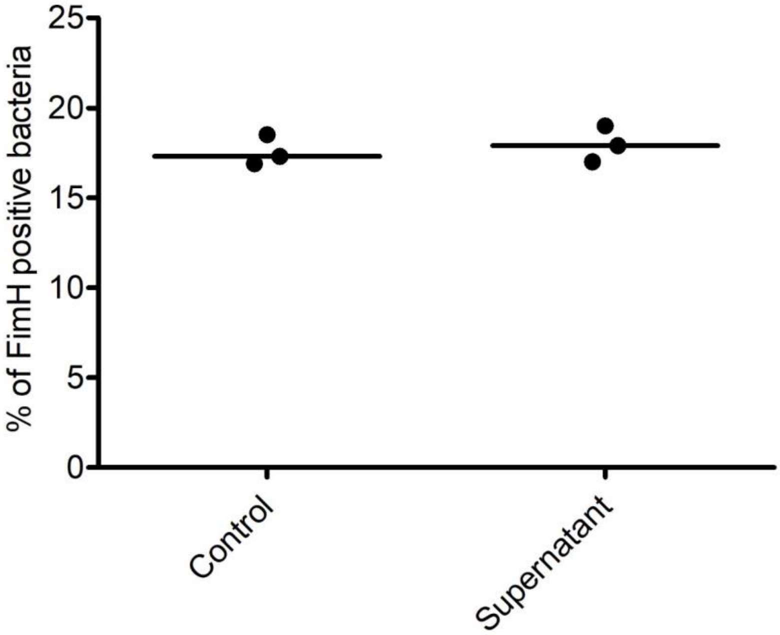

Supplementary figure 4.

A. Relative expression of *fimH* mRNA in *Salmonella* Typhimurium in supernatant above IPEC-J2 cells after 120 minutes of infection. Real-time RT-PCR was used to analyze the expression of *fimH* mRNA, and its levels were normalized against 16s RNA, and fold change was measured over the control infection. Data represent the mean±SD of three independent experiments. Triplicate samples were analyzed in each experiment to confirm the accuracy and reproducibility of qPCR. Statistical differences were analyzed by the t-student test. *P < 0.05, **P < 0.01, and ***P < 0.001.

B. Percent of FimH positive *Salmonella* Typhimurium in supernatant above IPEC-J2 cells after 120 minutes of infection measured by flow cytometry. Data represent the three individual values and a mean from three independent experiments. Statistical differences were analyzed by the t-student test. *P < 0.05, **P < 0.01, and ***P < 0.001.
