# Supplementary material for: Pre-Growth Culture Conditions Affect Type 1 Fimbriae-Dependent Adhesion of Salmonella
Source: Int J Mol Sci. 2020 Jun 12;21(12):4206. doi: 10.3390/ijms21124206 (PMC7352897; doi:10.3390/ijms21124206)
Supplement: Supplementary file 1 [file ijms-21-04206-s001.zip › Supplementary_Figure_1.pdf]

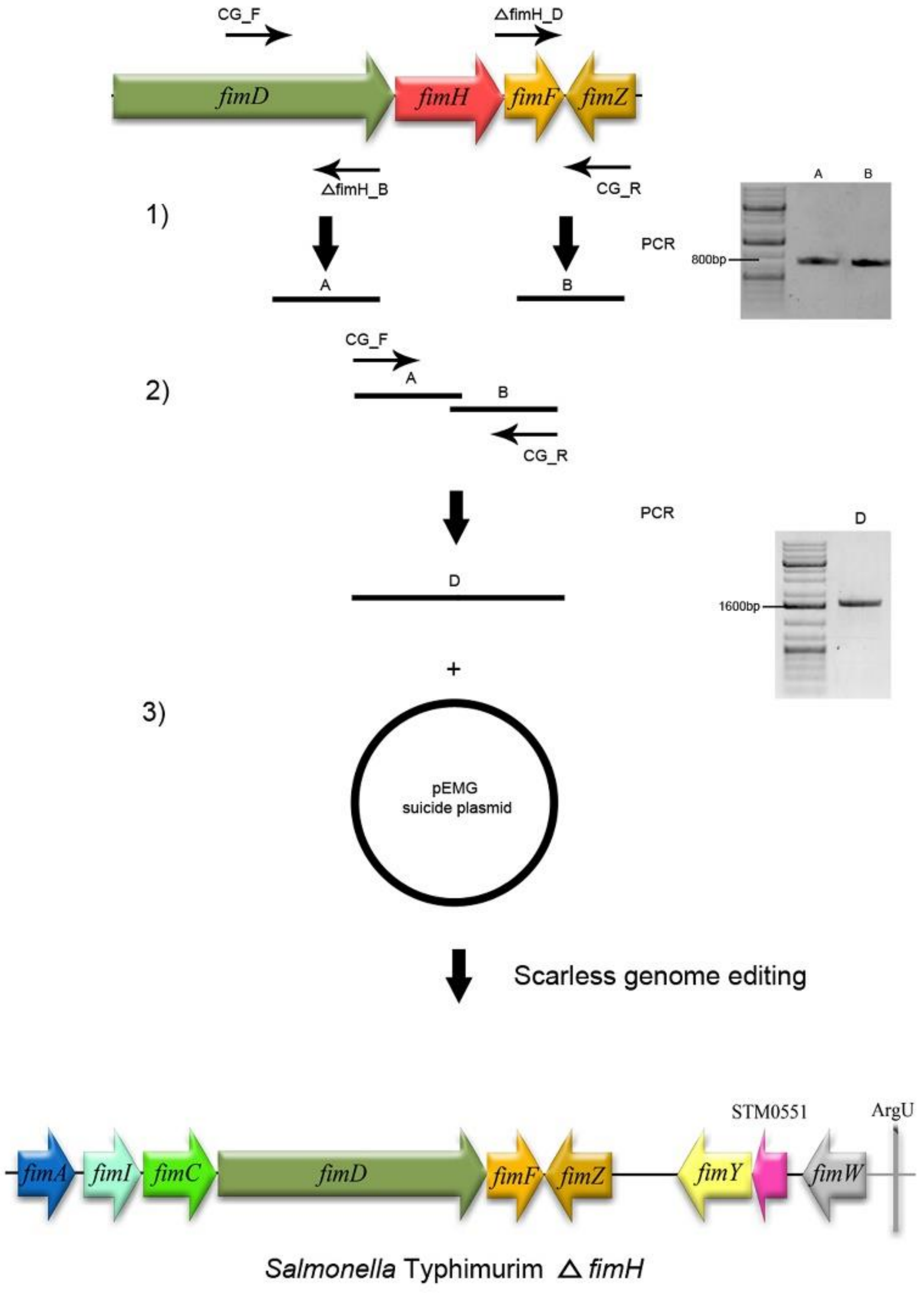

Supplementary figure 1: Schematic overview of *ΔfimH* suicide plasmid and *Salmonella* Typhimurium *ΔfimH* strain. DNA fragments A and B (800 bp) flanking the chromosome regions of interest were PCR amplified using CG_F, ΔFIMH_B, ΔFIMH_D and CG_R primers, fused by overlap extension PCR (fragment D) and inserted into the pEMG suicide plasmid.
